# Two-Dimensional RBF-ENO Method on Unstructured Grids

**DOI:** 10.1007/s10915-020-01176-2

**Published:** 2020-03-11

**Authors:** Jan S. Hesthaven, Fabian Mönkeberg

**Affiliations:** SB-MATH-MCSS, École Polytechnique Fédérale de Lausanne (EPFL), 1015 Lausanne, Switzerland

**Keywords:** Finite volume methods, Euler equations, High-order methods, Unstructured grids, Radial basis functions, 35L65, 65M08, 65D05, 65M12

## Abstract

Essentially non-oscillatory (ENO) and weighted ENO (WENO) methods on equidistant Cartesian grids are widely used to solve partial differential equations with discontinuous solutions. However, stable ENO/WENO methods on unstructured grids are less well studied. We propose a high-order ENO method based on radial basis function (RBF) to solve hyperbolic conservation laws on general two-dimensional grids. The radial basis function reconstruction offers a flexible way to deal with ill-conditioned cell constellations. We introduce a smoothness indicator based on RBFs and a stencil selection algorithm suitable for general meshes. Furthermore, we develop a stable method to evaluate the RBF reconstruction in the finite volume setting which circumvents the stagnation of the error and keeps the condition number of the reconstruction bounded. We conclude with several challenging numerical examples in two dimensions to show the robustness of the method.

## Introduction

Solving systems of hyperbolic conservation laws with high-order methods continues to attract substantial interest. In two dimensions, the system of conservation law on differential form is given as

1 $$\begin{aligned} \begin{aligned} u_t + f_1(u)_x + f_2(u)_y&= 0,&\mathbf{x} =(x,y) \in \mathbb {R}^2, t\in \mathbb {R}_{+},\\ u(\mathbf{x} ,0)&= u_0(\mathbf{x} ),&\mathbf{x} \in \mathbb {R}^2, \end{aligned} \end{aligned}$$

with the conserved variables $u:\mathbb {R}^2\times \mathbb {R}_{+} \rightarrow \mathbb {R}^N$, the flux $f_i:\mathbb {R}^N\rightarrow \mathbb {R}^N$ and the initial condition $u_0$. A well-known method to solve hyperbolic conservation laws is the class of finite volume methods. It is based on a discretization of the domain into control volumes and an approximation of the flux through its boundaries. Van Leer [33] introduced the MUSCL approach which is based on an high-order approximation of the flux through the boundaries. A well-known challenge for high-order methods is the property of the conservation laws to form discontinuities from smooth initial data [26]. Thus, solutions need to be defined in the weak (distributional) sense. To prevent stability issues, caused by the discontinuous solutions, Harten et al. [17] proposed the principle of essentially non-oscillatory (ENO) methods. A powerful extension of the ENO method is the weighted ENO (WENO) method [30]. Alternative methods to avoid stability problems and unphysical oscillations are based on adding artificial viscosity [34] or on the use of limiters [18]. A generalization of the finite volume method is the class of Discontinuous Galerkin (DG) finite element methods [7], for which it is necessary to add limiters to ensure non-oscillatory approximations [22]. There exist several approaches that combine RBFs with finite volume methods, e.g. a high-order WENO approach based on polyharmonics [1], a high-order WENO approach based on multiquadratics [6], a high-order RBF based CWENO method [21] and an entropy stable RBF based ENO method [20]. However, most of these are suitable only for one-dimensional grids or are at most second order accurate. We seek to overcome these limitations with a new RBF-ENO method on two dimensional general grids.

In Sect. 2, we introduce the finite volume scheme based on the MUSCL approach [33] and describe the basics of RBF interpolation in Sect. 3. Sections 4 and 5 contain the main contribution. In Sect. 4 we introduce a stable evaluation method for RBF interpolation with a polynomial augmentation, which circumvents the known error stagnation. In the same section we include a general proof of the order of convergence for RBFs augmented with polynomials. In Sect. 5, we introduce a smoothness indicator for RBFs, based on the sign-stable one-dimensional approach developed in [20]. We combine these results to construct an arbitrarily high-order RBF based ENO finite volume method. In Sect. 6, we demonstrate the robustness of the numerical scheme with a variety of numerical examples, while Sect. 7 offers a few concluding remarks.

## Finite Volume Methods

**Fig. 1:**
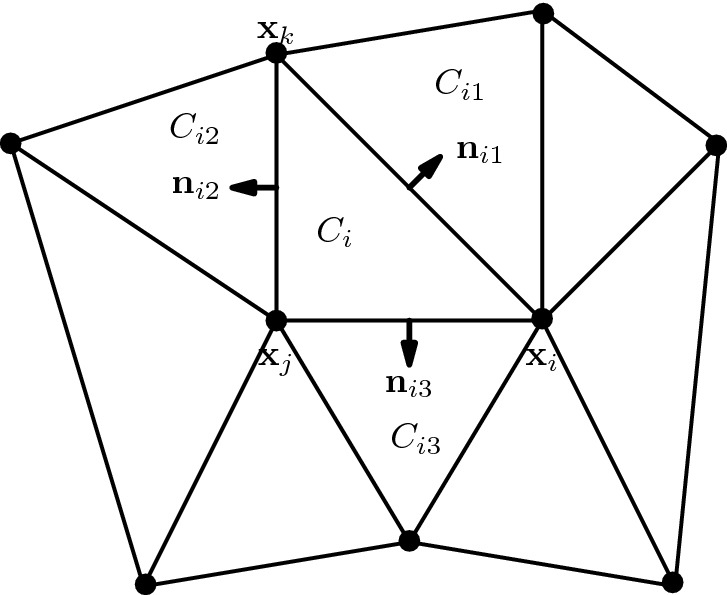
Triangulation for finite volume method

We assume a triangular grid of $\varOmega \subset \mathbb {R}^2$, consisting of triangular cells $C_i = (x_i ,x_k, x_j)$ as illustrated in Fig. 1. The finite volume method is based on cell averages $U_i = \frac{1}{|C_i|}\int _{C_i} u(x) \mathrm {d}x$ over the cell $C_i$. By integrating (1) over the cell and dividing it by its size $|C_i|$ we recover after applying the divergence theorem the semi-discrete scheme

2 $$\begin{aligned} \frac{\mathrm {d}U_i}{\mathrm {d}t} +\frac{1}{|C_i|}\sum _{l_{e}=1}^3 F_{il_{e}} = 0, \end{aligned}$$

with the numerical flux $F_{il_{e}} = F_{il_{e}}(U_i,U_{il_{e}},\mathbf{n }_{il_{e}})$ with the accuracy condition

3 $$\begin{aligned} \int _{S_{il_{e}}} \mathbf{f} (u)\cdot \mathbf{n }_{il_{e}}\mathrm {d}s(x)= F_{il_{e}}+ \mathcal {O}(\varDelta x^p), \end{aligned}$$

where $\mathbf{f} = (f_1,f_2)$, $S_{il_{e}} = \partial C_i \cap \partial C_{il_{e}}$, $U_{il_{e}}$ is the cell average of $C_{il_{e}}$ and $\mathbf{n }_{il_{e}}$ is the outward pointing normal vector. The numerical flux $F_{il_{e}}$ can be expressed using an (approximate) Riemann solver. A common choice is the Rusanov flux

4 $$\begin{aligned} F^{R}_{il_{e}}(U,V,{\mathbf{n }_{il_{e}}})= \frac{|S_{il_{e}}|}{2}\big (\mathbf{f} (U)+\mathbf{f} (V)\big )\cdot {\mathbf{n }_{il_{e}}}-\frac{{\alpha _{il_{e}}(U,V)}|S_{il_{e}}|}{2}\big (V - U\big ), \end{aligned}$$

with

5 $$\begin{aligned} {\alpha _{il_{e}}(U,V)} = \max \{ \lambda _{max}(\nabla _u\mathbf{f} (U)\cdot {\mathbf{n }_{il_{e}}}), \lambda _{max}(\nabla _u\mathbf{f} (V)\cdot {\mathbf{n }_{il_{e}}})\}. \end{aligned}$$

Here, $\lambda _{max}(A)$ is the biggest eigenvalue of *A* and $\mathbf{n }_{il_{e}}$ the normal vector to the interface $S_{il_{e}}$.

A high-order boundary integral approximation of (3) and a high-order accurate (polynomial) reconstruction *s* of the local solution can be used to evaluate the first order flux $F(U,V,{\mathbf{n }_{il_{e}}})$ on the quadrature points. This high-order flux can be written as

6 $$\begin{aligned} F_{il_{e}} = \sum _{k=1}^{n_Q} \omega _k F^R_{il_{e}}(s_i(\mathbf{x} _k),s_{il_{e}} (\mathbf{x} _k),\mathbf{n }_{il_{e}}), \end{aligned}$$

with the quadrature weights $\omega _k$, the quadrature points $\mathbf{x} _k$ for $k = 1,\dots ,n_Q$ with $n_Q\in \mathbb {N}$ the number of quadrature points and the high-order accurate reconstructions $s_i$ of the solution in cell $C_i$. The high-order reconstruction $s_i$ is based on a stencil of cells which includes $C_i$. In all cases, we can apply an arbitrary time discretization technique to recover a fully discrete scheme, e.g., an SSPRK method [14].

## Radial Basis Functions

The use of radial basis function for scattered data interpolation has a long history. Their mesh-free property and flexibility for high-dimensional data makes them advantageous when compared to polynomials.

### Standard Interpolation

Let us consider the interpolation problem $f|_X = (f(\mathbf{x} _1),\dots ,f(\mathbf{x} _n))^T\in \mathbb {R}^n$ on the scattered set of data points $X = (\mathbf{x} _1,\dots ,\mathbf{x} _n)^T$ with $\mathbf{x} _i\in \mathbb {R}^d$ for $f:\mathbb {R}^d\rightarrow \mathbb {R}$. We are seeking a function $s_{f,X}:\mathbb {R}^d\rightarrow \mathbb {R}$ such that

7 $$\begin{aligned} s_{f,X}(\mathbf{x} _i) = f(\mathbf{x} _i),\quad \hbox {for all }i = 1,\dots , n. \end{aligned}$$

The general radial basis function approximation is given as

8 $$\begin{aligned} s_{f,X}(\mathbf{x} ) = \sum _{i=1}^n a_i \phi (\varepsilon \Vert \mathbf{x} -\mathbf{x} _i\Vert ) + \sum _{j=1}^m b_j p_j(\mathbf{x} ), \end{aligned}$$

with polynomials $p_j\in \varPi _{l-1}(\mathbb {R}^d)$ the space of polynomials in $\mathbb {R}^d$ of order $l-1$, $l\in \mathbb {N}$, $m\in \mathbb {N}$ the degree of $\varPi _{l-1}(\mathbb {R}^d)$, a univariate continuous function $\phi$ (the radial basis function), the Euclidean norm $\Vert \cdot \Vert$ and the shape parameter $\varepsilon$. To ensure uniqueness of the coefficients $a_i$ and $b_j$ for all $i= 1,\dots ,n$ and $j=1,\dots , m$ we introduce the additional constraints

9 $$\begin{aligned} \sum _{i=1}^n a_i q(\mathbf{x} _i) = 0,\quad \hbox {for all } q\in \varPi _{l-1}(\mathbb {R}^d). \end{aligned}$$

To simplify the notation we write

10 $$\begin{aligned} \phi (\mathbf{x} -\mathbf{x} _i) := \phi (\varepsilon \Vert \mathbf{x} -\mathbf{x} _i\Vert ),\qquad \phi :\mathbb {R}^d\rightarrow \mathbb {R}. \end{aligned}$$

Finally, we express (7) and (9) by the system of equations

11 $$\begin{aligned} \begin{pmatrix} A &{} \quad P \\ P^T &{} \quad 0 \end{pmatrix} \begin{pmatrix} a\\ b \end{pmatrix} = \begin{pmatrix} f|_X\\ 0 \end{pmatrix}, \end{aligned}$$

with $A_{ij} = \phi (\mathbf{x} _i-\mathbf{x} _j)$, $P_{ij} = p_j(\mathbf{x} _i)$, $a = (a_1,\dots ,a_n)^T$ and $b = (b_1,\dots ,b_m)^T$. Depending on the choice of the RBF $\phi$ the polynomial term in (8) ensures the solvability of (11).

#### Definition 1

(*Conditionally positive function*) A function $\phi :\mathbb {R}^d\rightarrow \mathbb {R}$ is called conditionally positive (semi-) definite of order *m* if, for any pairwise distinct points $\mathbf{x} _1,\dots ,\mathbf{x} _n\in \mathbb {R}^d$ and $c = (c_1,\dots ,c_n)^T\in \mathbb {R}^n\setminus \{0\}$ such that

12 $$\begin{aligned} \sum _{i=1}^nc_ip(\mathbf{x} _i) = 0, \end{aligned}$$

for all $p\in \varPi _{l-1}(\mathbb {R}^d)$, the quadratic form

13 $$\begin{aligned} \sum _{j,k = 1}^n c_jc_k\phi (\mathbf{x} _j-\mathbf{x} _k), \end{aligned}$$

is positive (non-negative).

For a conditionally positive definite RBF $\phi$ of order *r* (11) has a unique solution if $\mathbf{x} _1,\dots ,\mathbf{x} _n$ are $\varPi _{l-1}(\mathbb {R}^d)$-unisolvent for $l\geqslant r$ [35]. A subclass of conditionally positive definite functions are the positive definite functions for which (13) holds but not (12).

Since the matrix *A* is positive definite for a positive definite function $\phi$, the existence of an unique solution to (11) is trivial for all $l\in \mathbb {N}$, if $\mathbf{x} _1,\dots ,\mathbf{x} _n$ are pairwise disjoint.

The most commonly used RBFs are listed in Table 1.

**Table 1:** Commonly used RBFs with $\mathbb {N}\not \ni \nu >0$, $k\in \mathbb {N}$ and $\varepsilon >0$

RBF	$\phi (r)$	Order
Infinitely smooth RBFs		
Multiquadratics	$(1+(\varepsilon r)^2)^\nu$	$\lceil \nu \rceil$
Inverse multiquadratics	$(1+(\varepsilon r)^2)^{-\nu }$	0
Gaussians	$\exp (-(\varepsilon r)^2)$	0
Piecewise smooth RBFs		
Polyharmonics	$r^{2k-d}$	*k*
	$r^{2k-d}\log (r)$	*k*

A well-known problem with RBFs is the ill-conditioning of the interpolation matrix and the resulting stagnation (saturation) of the error under refinement [9, 24]. One way to overcome the stagnation error is the augmentation with polynomials [4, 5, 12]. In this case, the polynomials take over the role for the interpolation and the RBFs ensure solvability of (11).

### Interpolation of Cell-Averages

The finite volume MUSCL approach is based on the interpolation of cell averages. Let us consider the interpolation problem $f|_S = (\bar{u}_1,\dots ,\bar{u}_n)^T\in \mathbb {R}^n$ on the stencil *S* of cells $C_1,\dots ,C_n$ in terms of the cell-averages $\bar{u}_1,\dots ,\bar{u}_n$. Based on (8) we have

14 $$\begin{aligned} s_{f,S}(x) = \sum _{i=1}^n a_i\lambda _{C_i}^{\xi }\phi (x-\xi ) + \sum _{j=1}^m b_j p_j(x), \end{aligned}$$

with $\lambda _{C}^{\xi } f$ being the average operator of *f* over the cell *C* with respect to the variable $\xi$ and $\{p_1,\dots ,p_m\}$ the polynomial basis of $\varPi _{l-1}(\mathbb {R}^d)$ [1]. Thus, we have the interpolation problem

15a $$\begin{aligned} \lambda _{C_j}s_{f,S}&= \bar{u}_j, \quad \hbox {for all } j = 1,\dots , n, \end{aligned}$$

15b $$\begin{aligned} \sum _{i=1}^n a_i\lambda _{C_i}(q)&= 0, \quad \hbox {for all } q\in \varPi _{l-1}(\mathbb {R}^d). \end{aligned}$$

The solvability of (15) is ensured provided $\phi$ is conditional positive definite in a pointwise sense and $\{\lambda _{C_i}\}_{i = 1}^n$ is $\varPi _{l-1}(\mathbb {R}^d)$-unisolvent [1].

## Stable RBF Evaluation for Fixed Number of Nodes

As mentioned in Sect. 3, the ill-conditioning of the RBF interpolation is a well-known challenge. However, RBFs within finite volume methods are of a slightly different nature. In general, the RBF approximation achieves exponential order of convergence for smooth functions by increasing the number of interpolation nodes in a certain domain. The setting for finite volume methods is different since the number of interpolation points remains fixed at a rather low number of nodes and only the fill-distance is reduced.

Based on [11, 12] it is known that the combination of polyharmonics and Gaussians with polynomials overcomes the stagnation error. Bayona [3] shows that under certain assumptions the order of convergence is ensured by the polynomial part.

We propose to use multiquadratic rather than polyharmonic or Gaussian RBFs to enable the use of the smoothness indicator, developed in [20]. Since the RBFs are only used to ensure solvability of the linear system, we can use

16 $$\begin{aligned} \varepsilon = \frac{1}{\varDelta x}, \end{aligned}$$

as the shape parameter with the separation distance $\varDelta x :=\min _{i\ne j}{\Vert \mathbf{x} _i-\mathbf{x} _j\Vert }$ for the interpolation nodes $\mathbf{x} _1,\dots , \mathbf{x} _n$ with $n\in \mathbb {N}$. To control the conditioning of the polynomial part we use the basis

17 $$\begin{aligned} p_i(x) = \tilde{p}_i(\varepsilon (\mathbf{x} -{\tilde{\mathbf{x}}})), \end{aligned}$$

for $i = 1,\dots , m$ with $\tilde{p}_i\in \{\mathbb {R}^d\rightarrow \mathbb {R}, \mathbf{x} \mapsto x_1^{\alpha _1}\dots x_d^{\alpha _d}| \; \sum _{i=1}^d \alpha _i < l, \alpha _i\in \mathbb {N}\}$, $\text {deg}(\tilde{p}_i) \leqslant \text {deg}(\tilde{p}_{i+1})$ and ${\tilde{\mathbf{x}}}\in \{\mathbf{x }_1\dots ,\mathbf{x }_n\}$. The best choice for ${\tilde{\mathbf{x}}}$ would be the barycenter of the stencil. However, to use the same polynomials for different stencils in the ENO scheme we choose the central one.

### Remark 1

The interpolation matrix is the same as the one with the interpolation basis $\tilde{p}_i$ with $i=1\dots ,m$, the RBFs with shape parameter 1, and the nodes $\tilde{\mathbf{x }}_1,\dots , \tilde{\mathbf{x }}_n$ with $\tilde{\mathbf{x }}_i = \varepsilon (\mathbf{x} _i-\mathbf{x} _1)$. This holds true for any $\varDelta x \rightarrow 0$ and $\varDelta \tilde{x} = 1$. Thus, the interpolation step in the finite volume method has the same condition number for all refinements as long as the interpolation nodes have a similar distribution.

### Stability Estimate for RBF Coefficients

In this section, we analyze the stability of the RBF interpolation based on (16) and (17) and show that the stability of the RBF coefficients depends only on the number of the interpolation nodes *n*. Then, for the one-dimensional case we show that the stability of the polynomial coefficients depends on *n* and the ratio of the maximum distance between the interpolation points *Dx* and the minimum distance $\varDelta x$. For higher dimension we conjecture that a similar result holds.

From [28] it follows that

#### Lemma 1

(Stability estimate [28]) For (11) there holds the stability estimate

18 $$\begin{aligned} \frac{\Vert \varDelta a\Vert _2}{\Vert a\Vert _2}\leqslant \frac{\lambda _{max}}{\lambda _{min}}\frac{\Vert \varDelta f\Vert _{2}}{\Vert f-P b\Vert _{2}}, \end{aligned}$$

with $\lambda _{min} := \inf _{a\ne 0, P^T a = 0} \frac{a^TA a}{a^T a}$ and $\lambda _{max}$ the maximal eigenvalue. Further, there exists an estimate for the polynomial coefficients

19 $$\begin{aligned} \frac{\Vert \varDelta b\Vert _2}{\Vert b\Vert _2}&\leqslant \frac{\lambda _{max,P^TP}}{\lambda _{min,P^TP}}\frac{\Vert P^T (\varDelta f - A\varDelta a)\Vert _{2}}{\Vert P^T ( f - A a)\Vert _{2}}, \end{aligned}$$

20 $$\begin{aligned}&\leqslant \Big (1+\frac{\lambda _{max}}{\lambda _{min}}\Big ) \frac{\lambda _{max,P^TP}}{\lambda _{min,P^TP}}\frac{\Vert P^T \varDelta f \Vert _{2}}{\Vert P^T ( f - A a)\Vert _{2}}, \end{aligned}$$

with the maximal and minimal eigenvalue of $P^TP$, $\lambda _{max,P^TP}$, $\lambda _{min,P^TP}$.

Thus, the stability of the method depends on the ratios

$$\begin{aligned} \lambda _{max}/ \lambda _{min} \quad \hbox {and}\quad \lambda _{max,P^TP}/ \lambda _{min,P^TP}. \end{aligned}$$

The maximal eigenvalues can be estimated by

21 $$\begin{aligned} \lambda _{max} = {\sup _{a\ne 0}} \frac{a^TA a}{a^T a} = \Vert A\Vert _{2}\leqslant \Vert A\Vert _{F} \leqslant n \max _{i,j}|A_{i,j}|. \end{aligned}$$

Note that $\lambda _{min}$ is not the smallest eigenvalue of A, but its definition is similar. Schaback [28] established the following lower bound

#### Lemma 2

(Lower bound of $\lambda _{min}$ [28]) Given an even conditionally positive definite function $\phi$ with the positive generalized Fourier transform $\hat{\phi }$. It holds that

22 $$\begin{aligned} \lambda _{min} \geqslant \frac{\varphi _0(M)}{2 \varGamma (d/2 + 1)}\Big (\frac{M}{2\sqrt{\pi }}\Big )^d, \end{aligned}$$

with the function

23 $$\begin{aligned} \varphi _0(r) := \inf _{\Vert \omega \Vert _2\leqslant 2r}\hat{\phi }(\omega ), \end{aligned}$$

for $$M>0$$ satisfying

24 $$\begin{aligned} M\geqslant \frac{12}{\varDelta x}\Big (\frac{\pi \varGamma ^2(d/2+1)}{9}\Big )^{1/(d+1)}, \end{aligned}$$

or

25 $$\begin{aligned} M\geqslant \frac{6.38 d}{\varDelta x}, \end{aligned}$$

and with

26 $$\begin{aligned} \varGamma (x) = \int _0^\infty t^{x-1}\exp (-t)\mathrm {d}t, \qquad {\text {Re}}(x) > 0. \end{aligned}$$

It remains to estimate $\varphi _0(M)$ depending on the RBFs. Some estimates for the common examples in Table 1 are

#### Lemma 3

(Estimate of $\varphi _0$ for multiquadratics [28]) Let $\phi$ be the multiquadratic RBF, then

27 $$\begin{aligned} \varphi _0(M) \geqslant \frac{\pi ^{d/2}\varGamma (d/2+\nu ) M^{-d-2\nu }\exp (-2M/\varepsilon )}{\varGamma (-\nu )}. \end{aligned}$$

Note that the lower bound of $\varphi _0$ of Lemma 3 is zero for $\nu \in \mathbb {N}$.

#### Lemma 4

(Estimate of $\varphi _0$ for Gaussians [35]) Let $\phi$ be the Gaussian RBF, then

28 $$\begin{aligned} \varphi _0(M) = (2\varepsilon ^2)^{-d/2}\exp (-M^2/\varepsilon ^2). \end{aligned}$$

#### Lemma 5

(Estimate of $\varphi _0$ for polyharmonics [35]) Let $\phi (r) = (-1)^{k+1}r^{2k}\log (r)$ be a polyharmonic RBF, then

29 $$\begin{aligned} \varphi _0(M) = (-1)^{k+1}2^{2k-1+d/2}\varGamma (k+d/2)k!(2M)^{-d-2k}. \end{aligned}$$

#### Corollary 1

By using the shape parameter (16) we recover

30 $$\begin{aligned} \frac{\Vert \varDelta a\Vert _2}{\Vert a\Vert _2}\leqslant {C(n,d)\Vert \varDelta f\Vert _2}, \end{aligned}$$

for all $\mathbf{x} _1,\dots ,\mathbf{x} _n$, $n\in \mathbb {N}$ and a constant *C*(*n*, *d*) which depends on the number of interpolation nodes *n* and the dimension *d*.

#### Proof

From Remark 1 we conclude

31 $$\begin{aligned} a := a(\mathbf{x} _1,\dots ,\mathbf{x} _n) = a(\tilde{\mathbf{x }}_1,\dots ,\tilde{\mathbf{x }}_n) =: \tilde{a}. \end{aligned}$$

From Lemmas 2 and 3 we obtain

32 $$\begin{aligned} \frac{\Vert \varDelta a\Vert _2}{\Vert a\Vert _2} = \frac{\Vert \varDelta \tilde{a}\Vert _2}{\Vert \tilde{a}\Vert _2} \leqslant {C(n,d,\varDelta \tilde{x})\Vert \varDelta f\Vert _2 = C(n,d,1)\Vert \varDelta f\Vert _2}, \end{aligned}$$

with a constant $C(n,d,\varDelta x)$ which depends on *n*, *d* and $\varDelta x$.

Hence, the stability of the RBF coefficients depends only on the number of interpolation nodes *n*. This analysis is dimension independent and it remains to estimate the ratio $\lambda _{max,P^TP}/ \lambda _{min,P^TP}$.

### Stability Estimate for Polynomial Coefficients

The analysis of the Gram matrix $G :=P^T P\in \mathbb {R}^{m\times m}$ is more challenging. For the polynomial basis (17) we have

33 $$\begin{aligned} G_{ij} = \sum _{l=1}^n p_i(\mathbf{x} _l)p_j(\mathbf{x} _l). \end{aligned}$$

We note that $P = \tilde{P}$ where $(\tilde{P})_{i,j} = \tilde{p}_i(\tilde{\mathbf{x }}_j)$ with $\tilde{\mathbf{x }}_j = \epsilon (\mathbf{x} _j - \mathbf{x} _1)$. In the one-dimensional case, the following estimate of the condition number holds for the Vandermonde matrix.

#### Lemma 6

(Conditioning of the Vandermonde matrix in one dimension, [13]) Let $V_n$ be the Vandermonde matrix $(V_n)_{i,j} = z_j^i$ with $z_i \ne z_j$ for $i\ne j$ and $z_j\in \mathbb {C}$. It holds that

34 $$\begin{aligned} \max _j\prod _{i\ne j}\frac{\max (1,|z_i|)}{|z_j-z_i|}<\Vert V_n^{-1}\Vert _{\infty }\leqslant \max _j\prod _{i\ne j}\frac{1+|z_i|}{|z_j-z_i|}. \end{aligned}$$

#### Corollary 2

35 $$\begin{aligned} \frac{\lambda _{max,P^TP}}{\lambda _{min,P^TP}} \leqslant \bigg (\frac{Dx}{\varDelta x}+1\bigg ) ^2\bigg (\frac{Dx}{\varDelta x}\bigg )^{2n}\frac{n^4}{\big (\left\lfloor n/2-1 \right\rfloor !\big )^4}, \end{aligned}$$

with $$Dx = \max _{i\ne j} |x_i-x_j|$$.

#### Proof

We start with the estimate of $\Vert P\Vert _{\infty }$

36 $$\begin{aligned} \Vert P\Vert _{\infty }&= \max _{i} \sum _{j=1}^n \Big (\frac{x_i-x_1}{\varDelta x}\Big )^{j-1} \leqslant \max _{i} \sum _{j=1}^n \Big (\frac{Dx}{\varDelta x}\Big )^{j-1} \leqslant \frac{\big (\frac{Dx}{\varDelta x}\big )^n -1}{\frac{Dx}{\varDelta x}-1},\end{aligned}$$

37 $$\begin{aligned}&\leqslant n \bigg (\frac{Dx}{\varDelta x}\bigg )^n, \end{aligned}$$

To estimate the norm of $P^{-1}$ we use Lemma 6

$$\begin{aligned} \Vert P^{-1}\Vert _{\infty }&\leqslant \max _{i}\prod _{j\ne i}\frac{1 + |\tilde{x}_j|}{|\tilde{x}_i - \tilde{x}_j|} = \max _{i}\prod _{j\ne i}\frac{\varDelta x +| {x}_j-x_1|}{|{x}_i - {x}_j|},\\&\leqslant \max _{i}\prod _{j\ne i}\frac{\varDelta x +Dx}{|j-i|\varDelta x}= \bigg (\frac{Dx}{\varDelta x}+1\bigg )\max _{i}\frac{1}{\prod _{j\ne i}|j-i|},\\&\leqslant \bigg (\frac{Dx}{\varDelta x}+1\bigg )\frac{1}{\prod _{j\ne \left\lfloor n/2 \right\rfloor }|j- \left\lfloor n/2 \right\rfloor |} \leqslant \bigg (\frac{Dx}{\varDelta x}+1\bigg )\frac{1}{\prod _{j<\left\lfloor n/2 \right\rfloor }|j|^2},\\&\leqslant \bigg (\frac{Dx}{\varDelta x}+1\bigg )\frac{1}{ \big (\left\lfloor n/2-1 \right\rfloor !\big )^2}. \end{aligned}$$

Furthermore, we have the standard estimate

38 $$\begin{aligned} \frac{1}{\sqrt{n}}\Vert A\Vert _{\infty } \leqslant \Vert A\Vert _{2}\leqslant \Vert A\Vert _{\infty }\sqrt{m}, \end{aligned}$$

for $A \in \mathbb {R}^{m\times n}$. From [32] we recover

39 $$\begin{aligned} \hbox {cond}_2 P^T P =( \hbox {cond}_2 P)^2, \end{aligned}$$

when $n = m$. Combined, this yields

40 $$\begin{aligned} \frac{\lambda _{max,P^TP}}{\lambda _{min,P^TP}} = \Vert P^{-1}\Vert _2 ^2\Vert P\Vert _{2}^2 \leqslant n^2 \Vert P^{-1}\Vert _{\infty }^2 \Vert P\Vert _{\infty }^2. \end{aligned}$$

Applying Corollary 2 to uniformly distributed nodes in $\mathbb {R}$ we obtain $Dx/\varDelta x = n-1$ and the condition number of $P^T P$ is uniformly bounded for all $\varDelta x$ by

41 $$\begin{aligned} \frac{\lambda _{max,P^TP}}{\lambda _{min,P^TP}} \leqslant \frac{(n-1)^{n}n^3}{\big (\left\lfloor n/2-1 \right\rfloor !\big )^2}. \end{aligned}$$

The proof of this estimate does not hold true for two-dimensional interpolation. However, we conjecture that similar bounds hold, as is confirmed in Table 2. Note that the reconstructions from (6) are based on a stencil in a grid. Thus, $Dx/\varDelta x$ is bounded for these interpolation problems.

**Table 2:** Comparison of maximum and minimum condition numbers arising for different polynomial degrees and different orders of MQs *k*

deg. poly.	1	2	2	3	3	3	4	4	4	4
*k*	1	1	2	1	2	3	1	2	3	4
min(Cond)	2.0e02	4.4e02	3.3e04	4.6e03	2.4e04	3.4e09	5.3e04	1.7e05	4.0e08	9.4e13
max(Cond)	2.7e02	7.8e02	1.1e05	8.7e03	7.1e04	6.8e09	4.2e05	2.0e06	1.8e09	9.4e14

### Approximation by RBF Interpolation Augmented with Polynomials

Considering ansatz (8) for the interpolation problem (7), (9) Bayona shows in [3], under the assumption of full rank of *A* and *P*, that the order of convergence is at least $\mathcal {O}(h^{l+1})$ based on the polynomial part. With similar techniques we can relax the assumptions of full rank of *A* by assuming $\varphi$ to be a conditionally positive definite RBF of order $l+1$.

#### Theorem 1

Let *f* be an analytic multivariate function and $\varphi$ a conditionally positive definite RBF of order $l+1$. Further, we assume the existence of a $\varPi _{l}(\mathbb {R}^d)$-unisolvent subset of *X*. It follows

42 $$\begin{aligned} \Vert s_{f,X} - f\Vert _{\infty } \leqslant \mathcal {O}(h^{l+1}). \end{aligned}$$

#### Proof

Let us consider $x_0\in \mathbb {R}^d$ where $x_0$ does not have to be a node. By the assumption that *f* is analytic, it admits a Taylor expansion in a neighborhood of $\mathbf {x}_0$

43 $$\begin{aligned} f(\mathbf {x}) = \sum _{k\geqslant 1} L_k[f(\mathbf {x}_0)] p_k(\mathbf {x}-\mathbf {x}_0), \end{aligned}$$

with $L_k[f(\mathbf {x}_0)]\in \mathbb {R}$ the coefficients for *f* around $\mathbf {x}_0$, e.g., $L_k[f(\mathbf {x}_0)] = \frac{1}{k!}f^{(k)}(\mathbf {x}_0)$ for univariate functions. Thus, we recover

44 $$\begin{aligned} f|_X = (f(\mathbf {x}_i))_{i=1}^n = \sum _{k\geqslant 1}L_k[f(\mathbf {x}_0)] \mathbf {p}_k, \end{aligned}$$

with $$\mathbf {p}_k =(p_k(\mathbf {x}_i-\mathbf {x}_0))_{i=1}^n.$$

Note that $\mathbf {a}_k\in \mathbb {R}^n$, $\mathbf {b}_k\in \mathbb {R}^m$ are given by

45 $$\begin{aligned} \begin{pmatrix} A &{} \quad P \\ P^T &{} \quad 0 \end{pmatrix} \begin{pmatrix} \mathbf {a}_k\\ \mathbf {b}_k \end{pmatrix} = \begin{pmatrix} \mathbf {p}_k\\ 0 \end{pmatrix}, \end{aligned}$$

and they satisfy

46 $$\begin{aligned} \begin{pmatrix} a\\ b \end{pmatrix} = \sum _{k\geqslant 1} L_k[f(\mathbf {x}_0)] \begin{pmatrix} \mathbf {a}_k\\ \mathbf {b}_k \end{pmatrix}. \end{aligned}$$

Since there exists a $\varPi _{l}(\mathbb {R}^d)$-unisolvent subset and by the well-posedness of (45), we have

47 $$\begin{aligned} a_{k,i} = 0, \qquad b_{k,j} = \delta _{k,j}, \end{aligned}$$

for $i = 1,\dots ,n$ and $j,k = 1,\dots ,m$. This allows us to write the interpolation function as

48 $$\begin{aligned} s_{f,X}(\mathbf {x})&= \sum _{i=1}^n a_i\phi (\mathbf {x}-\mathbf {x}_i) + \sum _{j=1}^m b_j p_j(\mathbf {x}),\end{aligned}$$

49 $$\begin{aligned}&=\sum _{k = 1}^m L_k[f(\mathbf {x}_0)] p_k(\mathbf {x}) + \sum _{k>m} \sum _{i=1}^n L_k[f(\mathbf {x}_0)] a_{k,i}\phi _i(\mathbf {x})\end{aligned}$$

50 $$\begin{aligned}&\quad +\, \sum _{k>m} \sum _{l=1}^s L_k[f(\mathbf {x}_0)] b_{k,l}p_l(\mathbf {x}), \end{aligned}$$

and recover

51 $$\begin{aligned} f(\mathbf {x}) - s_{f,X}(\mathbf {x})&= \sum _{k>m} L_k[f(\mathbf {x}_0)] p_k(\mathbf {x}) - \sum _{k>m} \sum _{i=1}^n L_k[f(\mathbf {x}_0)] a_{k,i}\phi _i(\mathbf {x})\nonumber \\&\quad -\, \sum _{k>m} \sum _{l=1}^m L_k[f(\mathbf {x}_0)] b_{k,l}p_l(\mathbf {x}) = r_m(\mathbf {x}) - s_{r_m}(\mathbf {x}). \end{aligned}$$

with $$r_m(\mathbf {x}) = \sum _{k>m} L_k[f(\mathbf {x}_0)] p_k(\mathbf {x})$$.

Given the estimate of DeMarchi et al. [8]

52 $$\begin{aligned} \Vert s_{f,X}\Vert _{\infty } \leqslant C (\Vert f\Vert _{\ell _\infty (X)} + \Vert f\Vert _{\ell _2 (X)}), \end{aligned}$$

we conclude

53 $$\begin{aligned} \Vert f-s_{f,X}\Vert _{\infty } = \Vert r_m-s_{r_m,X}\Vert _{\infty } \leqslant C h^{l+1}, \end{aligned}$$

with $$\Vert r_m\Vert _{\infty } \leqslant C h^{l+1}$$. $$\square $$

### Numerical Examples

In this section, we seek to verify the results in the finite volume setup (fixed number of interpolation nodes). Let $\varOmega = [0,1]^2$ and $f:\varOmega \rightarrow \mathbb {R}$ be a function and $\delta >0$. We approximate *f* by dividing the domain into subdomains of size $\delta \times \delta$ and solve in each subdomain the interpolation problem with *N* nodes given from an Halton sequence [16]. Since the condition number depends on the maximal distance divided by the separation distance $Dx/\varDelta x$, we use the Halton sequences with a separation distance bigger than $0.5\delta /\sqrt{N}$. We test the following functions

$$\begin{aligned} f_1(x,y)&= \sin (2\pi (x^2+2y^2))-\sin (2\pi (2x^2+(y-0.5)^2)),\\ f_2(x,y)&= \exp (-(x-0.5)^2-(y-0.5)^2),\\ f_3(x,y)&= \sin (2x)+\exp (-x),\\ f_4(x,y)&= 1+ \sin (4x)+\cos (3x)+\sin (2y). \end{aligned}$$

**Fig. 2:**
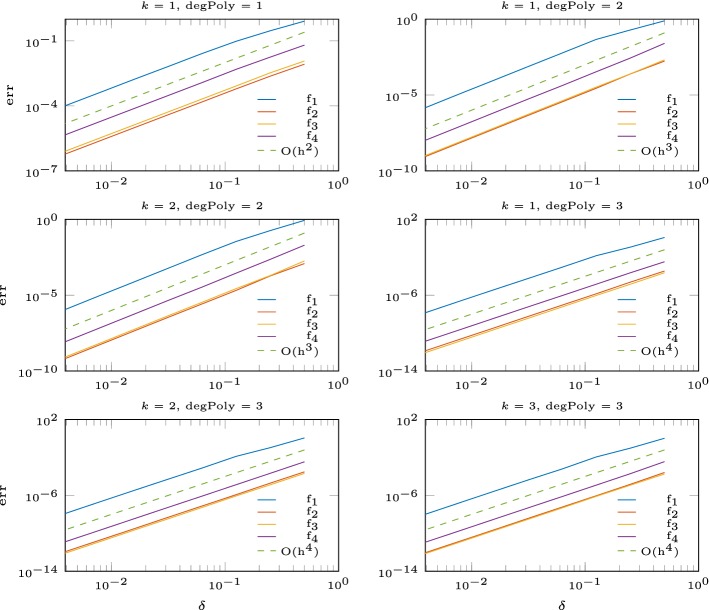
Error for the RBF interpolation with polynomial degree 1, 2, 3

**Fig. 3:**
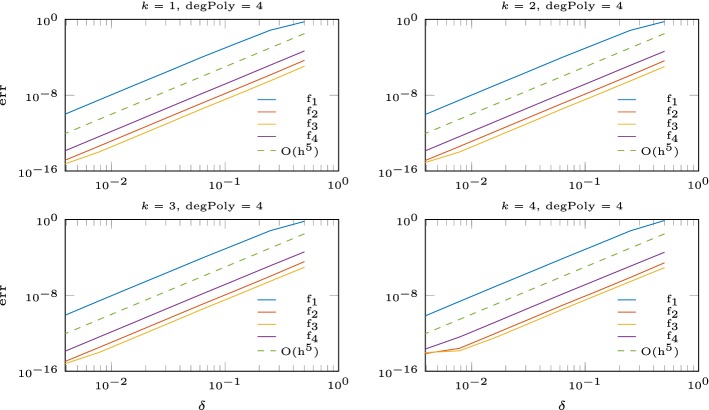
Error for the RBF interpolation with polynomial degree 4

In Figs. 2 and 3 we show the error of the interpolation problem and confirm the correct order of convergence for the multiquadratic interpolation augmented with a polynomial of degree *l* of order $k \leqslant {l}$. For polynomial degree ${l} = 4$ we observe that the convergence breaks down for $\delta < 2^{-7}$. This happens at small errors $\approx 10^{-15}$ and high condition numbers $>10^{13}$, as is shown in Table 2.

Furthermore, we verify the results from Sect. 4. Table 2 supports the conjecture that the condition number remains constant for a fixed number of interpolation nodes *n* and a fixed ratio $Dx/\varDelta x$.

We also observe that the condition number stays constant for the refined grids, and it is considerably smaller for first order multiquadratics $k=1$ than for the higher order ones.

## RBF-ENO Method

In this section, we introduce a new RBF-ENO method on two-dimensional general grids that can be generalized to higher dimensions. The method is based on the MUSCL approach described in Sect. 2, the RBF-ENO reconstruction introduced in [20], and the evaluation technique discussed in Sect. 4.

The finite volume method relies on the high-order flux (6) based on the boundary integral of the Rusanov flux (4) which is approximated by the Gauss-Legendre quadrature [2]. For the evaluation of the high-order flux we use the RBF reconstruction (14) and to compute the cell average we use a cubature rule for triangles [10]. The ENO reconstruction (Algorithm 1) is based on the one introduced by Harten et al. [17]. Thus, we recursively add one cell to the stencil $S_i$ and all its neighbors to a list of possible choices $N_i$ for the next step. In each step, we add the cell in $N_i$ which results in the stencil that has the smallest smoothness indicator *IS*, indicating the smoothness of the solution on a stencil. It is well-known that this strategy comes with high costs, but is also very robust. As the smoothness indicator we choose a generalization of the one-dimensional indicator introduced in [20]

54 $$\begin{aligned} IS_{RBF}(s) := \sum _{i=1}^n a_i^2, \end{aligned}$$

for the reconstruction $s(\mathbf{x} ) = \sum _{i=1}^n a_i\lambda _{C_i}^{\xi }\phi (\mathbf{x} -\xi ) + \sum _{j=1}^m b_j p_j(\mathbf{x} )$.
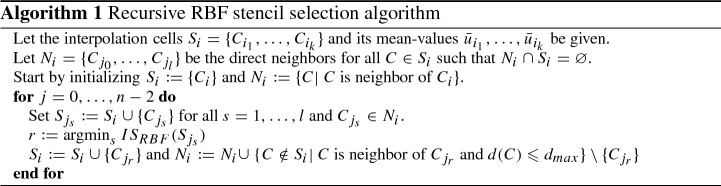


It is important to choose the right degree of the polynomial for each stencil. For a polynomial of degree *l* we need at least $n = \frac{(l+2)(l+1)}{2}$ cells, and thus $l = -1.5 + \frac{1}{2}\sqrt{1 + 8n}$. To reduce the probability of $\{\lambda _{C_{i_j}}\}_{j = 1}^n$ having no $\varPi _{l}(\mathbb {R}^d)$-unisolvent subset, we choose

55 $$\begin{aligned} l = {\left\{ \begin{array}{ll} \left\lfloor -2.5 + \frac{1}{2}\sqrt{1 + 8(n-1)} \right\rfloor , &{} \quad n\geqslant 5,\\ 0&{}\quad n <5. \end{array}\right. } \end{aligned}$$

Furthermore, we use multiquadratics with a shape parameter based on (16) and the polynomials (17). Since the order of convergence is not influenced by the order of the multiquadratics and following the observations in Sect. 4.4, we choose first order multiquadratics.

We need to slightly adapt the evaluation method from Sect. 4 to use it for the RBF-ENO method. The coefficients $a_i$ depend on the shape parameter. Thus, we must compare the smoothness indicator (54) with respect to the same shape parameter. By assuming approximately uniform equilateral triangles, we approximate $\varDelta x$ as

56 $$\begin{aligned} \varDelta x \approx \min _{j}2r_{j,inscr} \approx 2r_{i,inscr} \approx \sqrt{|C_i|}, \end{aligned}$$

with the radius $r_{j,inscr}$ of the inscribed circle of the *jth* cell and $|C_i|$ is the area of the *i*th cell. The last estimate comes from

57 $$\begin{aligned} |C_i| = 3\sqrt{3} r_{i,inscr}^2 \approx 4r_{i,inscr}^2, \end{aligned}$$

where we assume $C_i$ to be an equilateral triangle. Hence, we choose the shape parameter

58 $$\begin{aligned} \varepsilon = \frac{1}{\sqrt{|C_i|}}, \end{aligned}$$

with the polynomial basis (17).

The advantage of RBFs over polynomials is the ability to deal with a stencil with a variable number of elements. The condition for RBFs to have a well-defined system of equations is the existence of a subset which is $\varPi _{l}(\mathbb {R}^d)$-unisolvent and *l* must be larger than the order of the RBF. Thus, we can use a bigger stencil than the dimension of $\varPi _{l}(\mathbb {R}^d)$ to circumvent cell constellations that are ill-conditioned. To keep the stencil compact we classify each cell around the central one, depending on its distance $d\in \mathbb {N}$, such that

$$\begin{aligned} d(C) = 0,&\text{ if } C = C_i,\\ d(C) = 1,&\text{ if } C \text{ is } \text{ a } \text{ direct } \text{ neighbor } \text{ of } C_{i},\\ d(C) = 2,&\text{ if } C \text{ has } \text{ a } \text{ neighbor } \tilde{C} \text{ with } d(\tilde{C})=1,\\ \dots&\end{aligned}$$

and introduce $d_{max}$ as the maximal distance. A stable configuration for second to fourth order methods is given in Table 3.

**Table 3:** Stencil setting depending on the polynomial degree *l*

deg. poly. *l*	1	2	3
*n*	5	12	30
$d_{max}$	3	5	8

Note that (55) does not coincide with the values from Table 3. However, from numerical experiments this combination seems superior.

*Summary of the RBF-ENO method*

- Finite volume method with a high-order flux (6);
- The Gauss-Legendre quadrature [2] to approximate the boundary integral of the Rusanov flux (4);
- Reconstruction based on the RBF approach (14) with the polynomial basis (17);
- First order multiquadratics with shape parameter (58);
- Size *n* of stencil and $d_{max}$ from Table 3 depending on the order of the method;
- Stencil selection: Algorithm 1 and smoothness indicator (54) with polynomial degree (55).

## Numerical Results

In this chapter, we demonstrate the robustness of the second and third order RBF-ENO method on general grids. For the time discretization we use a third order SSPRK method [14]. The grids are generated by *distmesh*2*d*(), which is based on the Delaunay algorithm [27].

### Linear Advection Equation

We consider the linear advection equation in two dimensions

59 $$\begin{aligned} u_t + a u_x + b u_y= 0, \end{aligned}$$

with wave speed $a = 1$, $b = 0$ and periodic boundary conditions [26]. This results in a right moving wave given by the initial condition

60 $$\begin{aligned} u_0(x,y) = \cos (2\pi x)\cos (2\pi y) +10. \end{aligned}$$

**Fig. 4:**
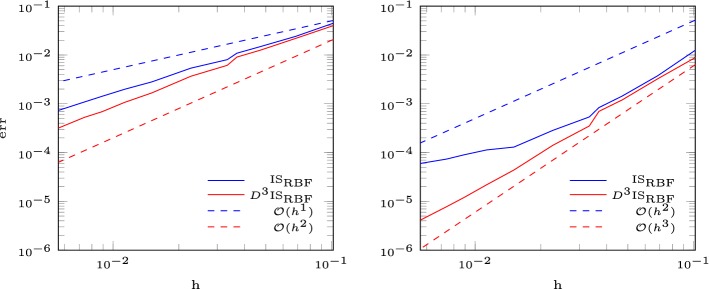
Error for the RBF-ENO method for the linear advection equation in 2D (left 2nd order, right 3rd order)

Figure 4 shows the error at $T = 0.1$. We observe a drop of the order of convergence after a certain level of refinement which is a known phenomena [19, 31]. This arises from constantly switching the stencil. For a very smooth function we recover the right order of convergence by multiplying the smoothness indicator with a penalty term $D^3$ which depends on the distance to the central cell

61 $$\begin{aligned} D := \frac{1}{|C_i|} \sum _{j\in S_i}\Vert x_{c,j}-x_{c,i}\Vert ^2, \end{aligned}$$

with the center $x_{c,i}$ of cell $C_i$. This gives preference to the central stencil.

### Burgers’ Equation

Next, we consider Burgers equation

62 $$\begin{aligned} u_t +\frac{1}{2}(u^2)_x + \frac{1}{2}(u^2)_y = 0, \end{aligned}$$

on the domain $\varOmega = [0,1]^2$ with the initial conditions

63 $$\begin{aligned} u_0 = {\left\{ \begin{array}{ll} -1 &{} \quad \hbox {if } x>0.5, \; y>0.5 , \\ -0.2&{} \quad \hbox {if } x< 0.5, \; y>0.5, \\ 0.5 &{} \quad \hbox {if } x< 0.5, \; y< 0.5, \\ 0.8 &{} \quad \hbox {if } x>0.5, \; y < 0.5. \\ \end{array}\right. } \end{aligned}$$

**Fig. 5:**
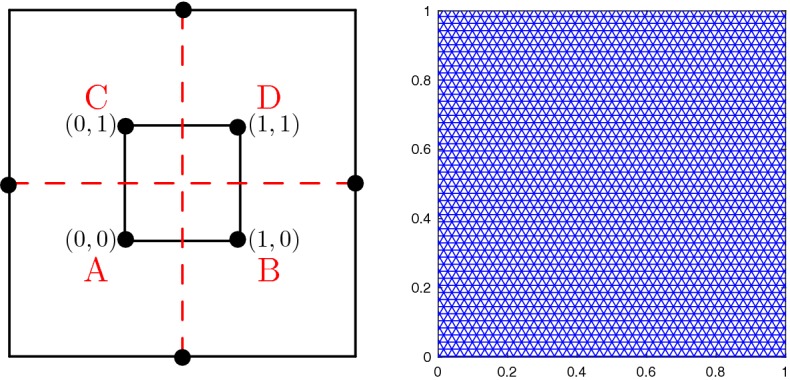
Domain extension, initial value composition and grid

**Fig. 6:**
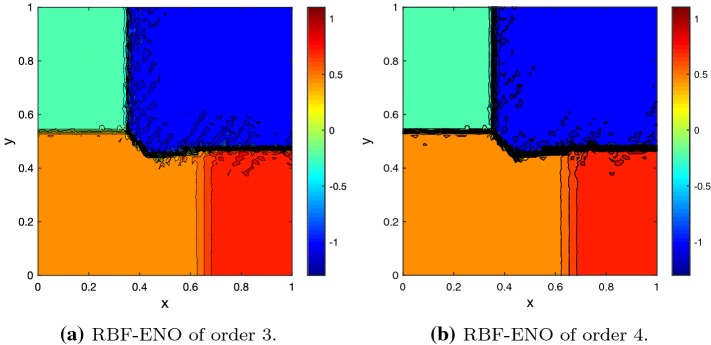
Solution of the Burgers’ equation at $T = 0.25$ with $N = 37{,}444$ cells, ${\text {CFL}}= 0.5$

The Burgers equation illustrates the behavior of the scheme with a non-linear flux and its ability to deal with discontinuities. Furthermore, the results can be compared with the exact solution [15]. The solution consists of shocks and rarefaction waves as its one-dimensional counterpart. To avoid boundary effects we increase the computational domain to $\varOmega = [-1,2]^2$ and keep the initial conditions for the extended square, see Fig. 5. The solutions at time $T=0.25$ for the 3rd and 4th order method are as expected, Fig. 6. There are some minor oscillations, but they remain small.

### KPP Rotating Wave

We consider the two-dimensional KPP rotating wave problem

64 $$\begin{aligned} u_t + (\sin (u))_x + (\cos (u))_y = 0, \end{aligned}$$

in the domain $\varOmega = [-2,2]^2$ with periodic boundary conditions and the initial conditions

65 $$\begin{aligned} u_0 = {\left\{ \begin{array}{ll} 3.5\pi &{} \quad \hbox {if } x^2+y^2 \leqslant 1, \\ 0.25\pi &{}\quad \hbox {otherwise } . \end{array}\right. } \end{aligned}$$

**Fig. 7:**
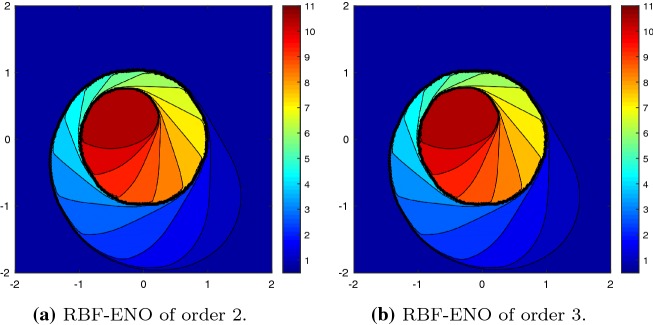
KPP problem at $T = 1$ with $N = 58{,}646$ cells, ${\text {CFL}}= 0.5$

This is a complex non-convex scalar conservation law [23]. The KPP problem was designed to test various schemes for entropy violating solutions. At time $T=1$ the solution forms a characteristic spiral, which is well-resolved for the second and third order method, as shown in Fig. 7.

### Euler Equations

Let us consider the two-dimensional Euler equations

66 $$\begin{aligned} \begin{pmatrix} \rho \\ m_1\\ m_2\\ E \end{pmatrix}_t + \begin{pmatrix} m_1\\ \frac{m_1^2}{\rho } + p\\ \frac{m_1 m_2}{\rho } \\ \frac{m_1}{\rho }(E + p) \end{pmatrix}_x + \begin{pmatrix} m_2\\ \frac{m_1 m_2}{\rho }\\ \frac{m_2^2}{\rho } + p\\ \frac{m_2}{\rho }(E + p) \end{pmatrix}_y= 0, \end{aligned}$$

with the density $\rho$, the mass flux $m_1$ and $m_2$ in *x*- and *y*-direction, the total energy *E*, and the pressure

67 $$\begin{aligned} p= (\gamma -1)\Big (E-\frac{(m_1^2 + m_2^2)}{2\rho }\Big ). \end{aligned}$$

The mass flux is given by $m = \rho u$. Further, we choose $\gamma = 1.4$ which reflects a diatomic gas such as air.

#### Isentropic Vortex

**Fig. 8:**
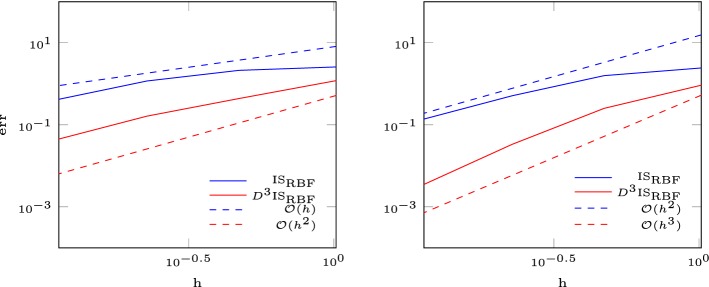
Error for the RBF-ENO method for the isentropic vortex problem. Left 2nd order, right 3rd order

The isentropic vortex problem describes the evolution of a inviscid isentropic vortex in a free stream on the domain $\varOmega = [-5,5]^2$. Proposed by Yee et al. [37] it is one of the few problems of the Euler equations with an exact solution. The initial conditions are

$$\begin{aligned} \rho&= \Big [1-\frac{\beta ^2(\gamma -1)}{8\gamma \pi ^2}\exp (1-r^2)\Big ]^{\frac{1}{(\gamma -1)}},\qquad u_1 = M\cos (\alpha )-\frac{\beta (y-y_c)}{2\pi }\exp \left( \frac{1-r^2}{2}\right) ,\\ u_2&= M\sin (\alpha )-\frac{\beta (x-x_c)}{2\pi }\exp \left( \frac{1-r^2}{2}\right) , \qquad r = \sqrt{(x-x_c)^2+(y-y_c)^2}, \end{aligned}$$

with the initial vortex strength $\beta$, the initial vortex center $(x_c,y_c)$ and periodic boundary conditions. The pressure is initialized by $p = \rho ^\gamma$ and $\alpha$ prescribes the passive advection direction. The exact solution is the initial condition propagating with speed *M* in direction $(\cos (\alpha ),\sin (\alpha ))$. The parameters are chosen as $M = 0.5$, $\alpha = 0$, $\beta = 5$ and $(x_c,y_c) = (0,0)$. We analyze the order of convergence at time $T = 1$. In Fig. 8 we observe the same behavior as for the linear advection equation. Again, we overcome this stability issue by introducing a penalty term $D^3$ which depends on the distance of the cell to its central one (61), and recover the optimal order of convergence.

#### Riemann Problem

The initial values for Riemann problems in two dimensions are constant in each quadrant

68 $$\begin{aligned} u_0 = {\left\{ \begin{array}{ll} (\rho _A,m_{1,A},m_{2,A},E_A) &{}\quad \hbox { if } x< 0.5, \; y< 0.5, \\ (\rho _B,m_{1,B},m_{2,B},E_B)&{} \quad \hbox { if } x>0.5, \; y< 0.5, \\ (\rho _C,m_{1,C},m_{2,C},E_C) &{} \quad \hbox { if } x< 0.5, \; y>0.5, \\ (\rho _D,m_{1,D},m_{2,D},E_D) &{} \quad \hbox { if } x>0.5, \; y>0.5, \\ \end{array}\right. } \end{aligned}$$

with the physical domain $\varOmega = [0,1]^2$, which is enlarged to $\varOmega = [-1,2]^2$ to reduce boundary effects. The values are chosen in such a way that only a single elementary wave appears at each interface. This results in 19 genuinely different configuration for a polytropic gas [25]. We test two of them, see Table 4.

**Table 4:** Initial values of the Riemann problem

	Riemann problem 4				Riemann problem 12			
	$\rho$	$u_1$	$u_2$	*p*	$\rho$	$u_1$	$u_2$	*p*
A	1.1	0.8939	0.8939	1.1	0.8	0	0	1
B	0.5065	0	0.8939	0.35	1	0	00.7276	1
C	0.5065	0.8939	0	0.35	1	0.7276	0	1
D	1.1	0	0	1.1	0.5313	0	0	0.4

**Fig. 9:**
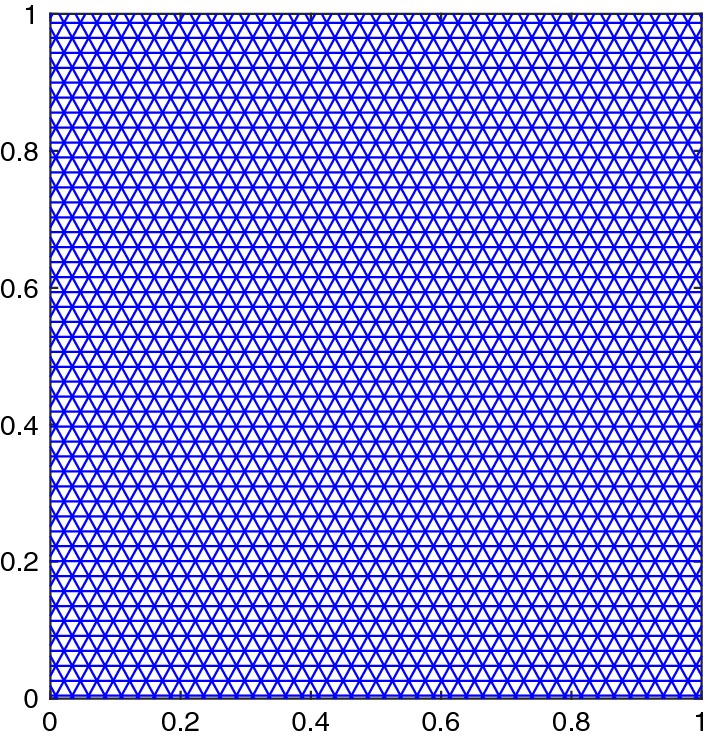
Grid for the Riemann problems

**Fig. 10:**
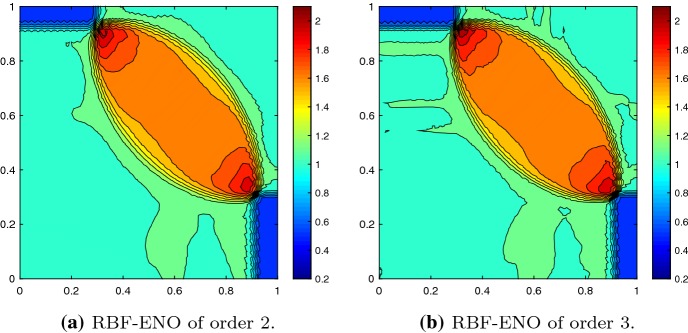
Riemann problem 4 at $T = 0.25$ with $N = 32{,}946$ cells in the extended domain, ${\text {CFL}}= 0.5$

**Fig. 11:**
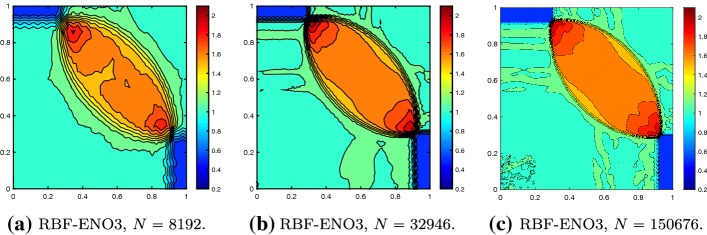
Convergence in *h* of the Riemann problem 4 at $T = 0.25$ with ${\text {CFL}}= 0.5$

We solve the Riemann problems until time $T = 0.25$ on the grid shown in Fig. 9. In Fig. 10 we see that the results of the 4th configuration are well resolved with the 2nd and 3rd order methods, while keeping the oscillations small. Furthermore, Fig. 11 illustrates the convergence in *h* for the RBF-ENO method of order 3.

**Fig. 12:**
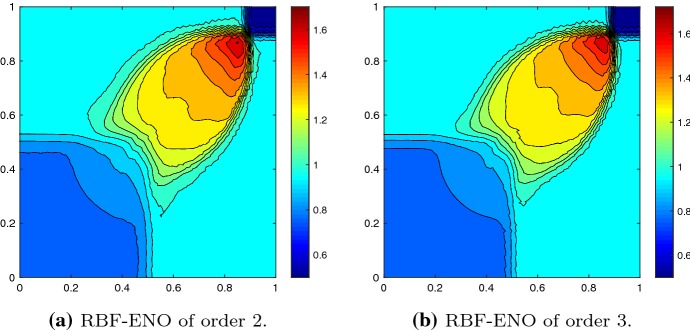
Riemann problem 12 at $T = 0.25$ with $N = 32{,}946$ cells in the extended domain, ${\text {CFL}}= 0.5$

**Fig. 13:**
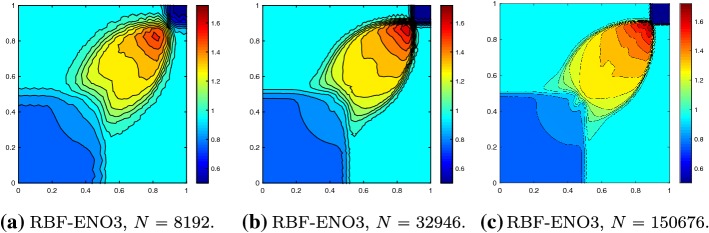
Convergence in *h* of the Riemann problem 12 at $T = 0.25$ with ${\text {CFL}}= 0.5$

For the Riemann problem 12 at time $T = 0.25$ the results are of a similar quality, see Figs. 12 and 13.

#### Shock Vortex Interaction

The shock vortex interaction problem was introduced to test high order methods [29]. It describes the interaction of a right-moving vortex with a left-moving shock in the domain $\varOmega = [0,1]^2$. The initial condition is given by the shock discontinuity

69 $$\begin{aligned} (\rho ,m_{1},m_{2},E) = {\left\{ \begin{array}{ll} (\rho _L,m_{1,L},m_{2,L},E_L) &{} \quad \hbox {if } x< 0.5, \\ (\rho _R,m_{1,R},m_{2,R},E_R)&{} \quad \hbox {if } x\geqslant 0.5, \\ \end{array}\right. } \end{aligned}$$

with the left state superposed by the perturbation

$$\begin{aligned} \delta u_1&= \epsilon \frac{y-u_c}{r_c}\exp (\beta (1-r^2)),&\delta u_2 = -\epsilon \frac{x-x_c}{r_c}\exp (\beta (1-r^2)),\\ \delta \theta&= -\frac{\gamma -1}{4\beta \gamma }\epsilon ^2\exp (2\beta (1-r^2)),&\delta s = 0, \end{aligned}$$

with the temperature $\theta = p/ \rho$, the physical entropy $s = \log p-\gamma \log \rho$ and the distance $r^2 = ((x-x_c)^2+(y-y_c)^2)/r_c^2$. The left state is given by $(\rho _L,u_{1,L},u_{2,L},E_L) = (1,\sqrt{\gamma },0,1)$ and the right state by

$$\begin{aligned} p_R&= 1.3,&\rho _R = \rho _L\Big (\frac{\gamma -1 + (\gamma +1)p_R}{\gamma +1 + (\gamma -1)p_R}\Big ),\\ u_{1,R}&= \sqrt{\gamma }+\sqrt{2}\Big (\frac{1-p_R}{\sqrt{\gamma -1+p_R(\gamma +1)}}\Big ),&u_{2,R} = 0. \end{aligned}$$

**Fig. 14:**
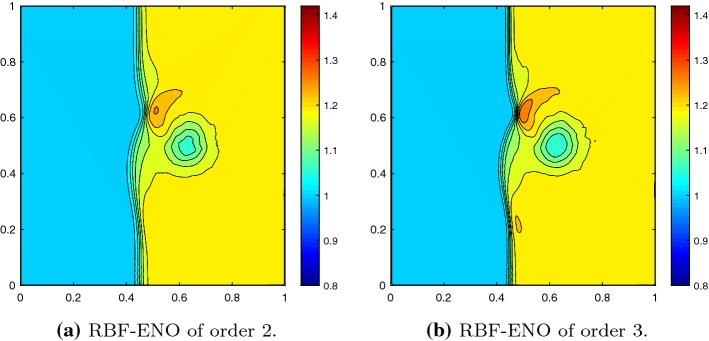
Shock vortex interaction problem at $T = 0.35$ with $N = 14{,}616$ cells, ${\text {CFL}}= 0.5$

**Fig. 15:**
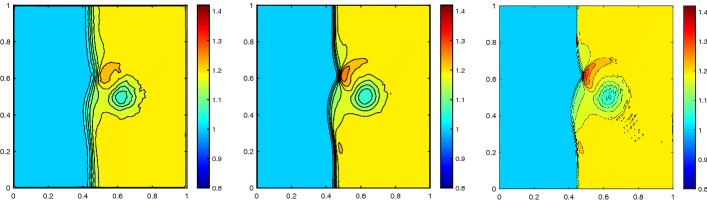
Convergence in *h* of the shock vortex interaction problem at $T = 0.35$ with ${\text {CFL}}= 0.5$

The parameter of the vortex are chosen as $\epsilon = 0.3$, $r_c = 0.05$, $\beta = 0.204$ with the initial center of the vortex $(x_c,y_c) = (0.25,0.5)$. Figure 14 shows the result of the second and third order RBF-ENO method at the final time $T = 0.35$ for $N = 14{,}616$ cells. The higher resolution of the third order method is clear. In Fig. 15 we see the convergence of the scheme for increasing number of cells. We observe minor oscillations for $N = 58{,}646$, but they remain stable.

#### Double Mach Reflection

**Fig. 16:**
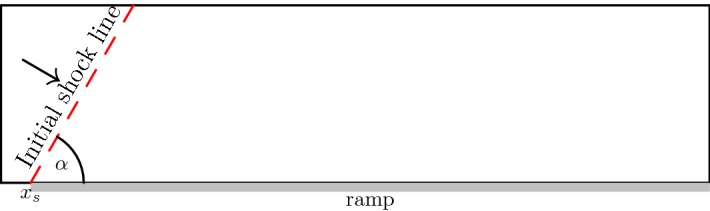
Domain for the double Mach reflection problem

The double Mach reflection problem is a standard benchmark for Euler codes that tests its robustness in the presence of a strong shock. It was introduced by Woodward et al. [36] and consists of a Mach 10 shock propagating at an angle of $30^\circ$ ($\alpha = 60^\circ$) into the ramp, see Fig. 16. The domain $\varOmega = [0,4]\times [0,1]$ contains a ramp starting at $x_s = 1/6$. As boundary conditions we have on the left side and on the ground in front of the ramp inflow boundary conditions with the post-shock values. On the ramp we use slip-wall conditions, on the top we apply the exact time dependent shock location and on the right outflow boundary conditions with the pre-shock conditions. The solution is simulated until $T = 0.2$ with the initial condition

70 $$\begin{aligned} (\rho ,m_{1},m_{2},E) = {\left\{ \begin{array}{ll} (8.0,57.1597,-33.0012,563.544) &{} \quad \hbox {post-shock} , \\ (1.4, 0,0,2.5)&{} \quad \hbox {pre-shock}. \end{array}\right. } \end{aligned}$$

**Fig. 17:**
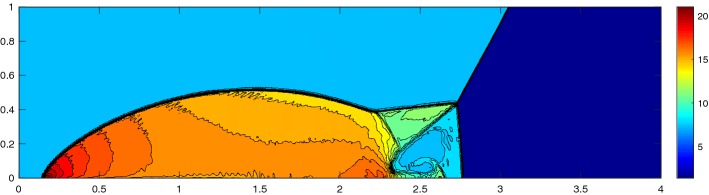
Double Mach reflection problem at $T = 0.2$ with $N = 151{,}216$ cells, ${\text {CFL}}= 0.5$ solved with the RBF-ENO of order 3

**Fig. 18:**
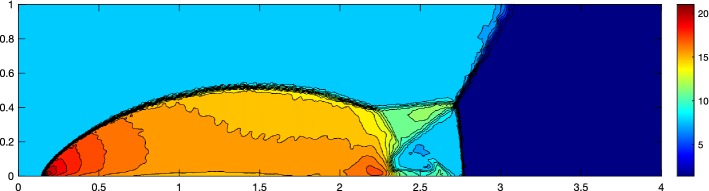
Double Mach reflection problem at $T = 0.2$ with $N = 41{,}140$ cells, ${\text {CFL}}= 0.5$ solved with the RBF-ENO of order 3 on totally unstructured grid

**Fig. 19:**
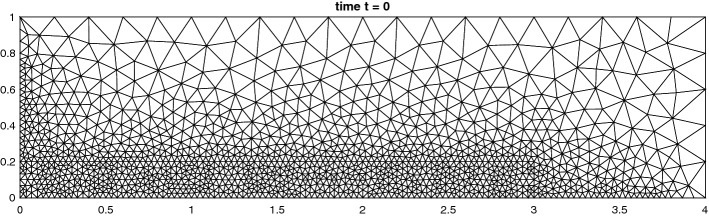
Example of totally unstructured grid with $N = 2843$ cells

To solve the double Mach reflection problem we must choose the multiquadratics of order *l* for a method of order *l* to get a stable solution, shown in Fig. 17. This suggests that the proposed stencil selection algorithm in [20] is more stable than just using a first order RBF in the same algorithm. To highlight the ability to deal with fully unstructured grids, we present a solution with around a quarter of the cells refined in the lower fifth of the domain, Fig. 18. The solution is based on a grid of the form of Fig. 19 with approximately six times more cells at each face. Note that the cells in the lower part have around the same size as the ones in the example from Fig. 17.

## Conclusions

In this work, we propose a new RBF-ENO method for multi-dimensional problems on general grids. We introduced a stable evaluation method for RBFs, augmented with polynomials and a stencil selection algorithm based on [20]. We showed that the algorithm preserves the expected accuracy and we demonstrated its robustness for challenging test cases, including two classic Riemann problems, the shock-vortex interaction and the double Mach reflection problem.

However, it is well-known that the strategy of the stencil selection algorithm is coming with high costs. As shown for the Burgers equation the method is working also in the 4th order setup, but due to the high number of cells in the stencil it is extremely costly.

In the future, we will combine the stable and flexible RBF-ENO method with the standard (polynomial) WENO method on structured grids, to offset some of the computational cost of the RBF-ENO scheme.
